# P-1973. Signal Detection of Neurotoxicity Associated with Carbapenem Use: A FAERS-Based Disproportionality Analysis

**DOI:** 10.1093/ofid/ofaf695.2140

**Published:** 2026-01-11

**Authors:** Linta Susan Kuriakose, Albin C Sebastian, Alvin Sunny

**Affiliations:** Square Hospital, West Panthapath, Dhaka, Bangladesh; Square Hospital, West Panthapath, Dhaka, Bangladesh; Square Hospital, West Panthapath, Dhaka, Bangladesh

## Abstract

**Background:**

Carbapenems are broad-spectrum β-lactam antibiotics widely used for treating severe and resistant infections. However, concerns regarding neurotoxicity—including seizures, encephalopathy, and altered mental status—have emerged, particularly in patients with renal impairment or central nervous system comorbidities. This study aimed to evaluate neurotoxicity signals associated with carbapenem use using the U.S. Food and Drug Administration’s Adverse Event Reporting System (FAERS) database through disproportionality analysis.

**Figure:**
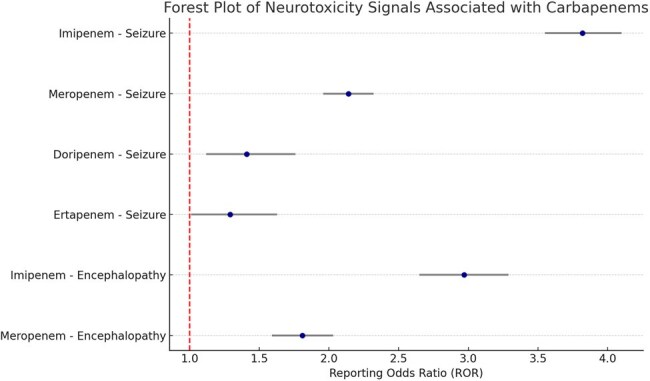
Forest Plot of Neurotoxicity Signals Associated with Carbapenems This forest plot presents the reporting odds ratios (RORs) and 95% confidence intervals for neurotoxicity adverse events related to carbapenem use, derived from the FAERS database. Notably, imipenem and meropenem show strong disproportionality signals for seizures and encephalopathy, indicating a potential safety concern requiring clinical vigilance, especially in high-risk populations.

**Methods:**

A retrospective pharmacovigilance study was conducted using FAERS reports from January 2010 to December 2023. Individual carbapenems (imipenem, meropenem, ertapenem, doripenem) were evaluated. Neurotoxicity-related Preferred Terms (PTs) were extracted using the Medical Dictionary for Regulatory Activities (MedDRA), including seizures, status epilepticus, confusion, hallucination, encephalopathy, and myoclonus. Disproportionality was assessed using Reporting Odds Ratio (ROR) and Proportional Reporting Ratio (PRR), with a signal defined as ROR lower 95% CI >1 and ≥3 reports.

**Results:**

A total of 5,632 neurotoxicity-related reports were associated with carbapenem use. Imipenem had the highest ROR for seizures (ROR: 3.82, 95% CI: 3.55–4.10; PRR: 3.21), followed by meropenem (ROR: 2.14, 95% CI: 1.96–2.32). Doripenem and ertapenem showed weaker but consistent signals. Encephalopathy was significantly associated with both imipenem (ROR: 2.97) and meropenem (ROR: 1.81). Combination therapy with valproate or cefepime showed increased signal amplification. Subgroup analysis revealed stronger associations in patients aged >65 and in those with renal dysfunction (reported in co-medications).

**Conclusion:**

This FAERS-based disproportionality analysis identified strong neurotoxicity signals associated with imipenem and meropenem, particularly for seizures and encephalopathy. These findings reinforce the need for dose adjustment, close neurologic monitoring, and cautious use in elderly patients or those with renal impairment. Further prospective studies are needed to validate causality and elucidate mechanisms.

**Disclosures:**

All Authors: No reported disclosures

